# Complete genome sequence of seed-transmitted soybean yellow mottle mosaic virus from soybeans in Korea

**DOI:** 10.1128/MRA.00837-23

**Published:** 2023-10-31

**Authors:** Sangmin Bak, Mina Kwon, Seungbin Baek, Minjoo Yang, Hong-Kyu Lee, Su-Heon Lee

**Affiliations:** 1 School of Applied Biosciences, Kyungpook National University, Daegu, South Korea; 2 Department of Applied Biology, Kyungpook National University, Daegu, South Korea; 3 Department of Plant Medicine, Kyungpook National University, Daegu, South Korea; 4 Institute of Plant Medicine, Kyungpook National University, Daegu, South Korea; Berkeley Institute for Data Science (BIDS), Berkeley, California, USA

**Keywords:** soybean yellow mottle mosaic virus, seed transmission, soybean, complete genome squence

## Abstract

Soybean yellow mottle mosaic virus (SYMMV), a member of the genus *Gammacarmovirus*, remains poorly understood in terms of its transmission pathway. This study reveals the complete genome sequence of a seed-transmitted isolate, ST-HB56, contributing to the understanding of SYMMV’s ecological dynamics.

## ANNOUNCEMENT

Soybean yellow mottle mosaic virus (SYMMV), a member of the genus *Gammacarmovirus*, which belongs to the family *Tombusviridae*, is classified in the recently renamed species *Gammacarmovirus glycinis*. Since the virus was initially described in Korean soybeans (1), it has been reported in several countries. Recent studies have provided confirmation that SYMMV occurs in early-stage soybeans (2) and is transmitted through seeds in certain legumes (3).

**Fig 1:**
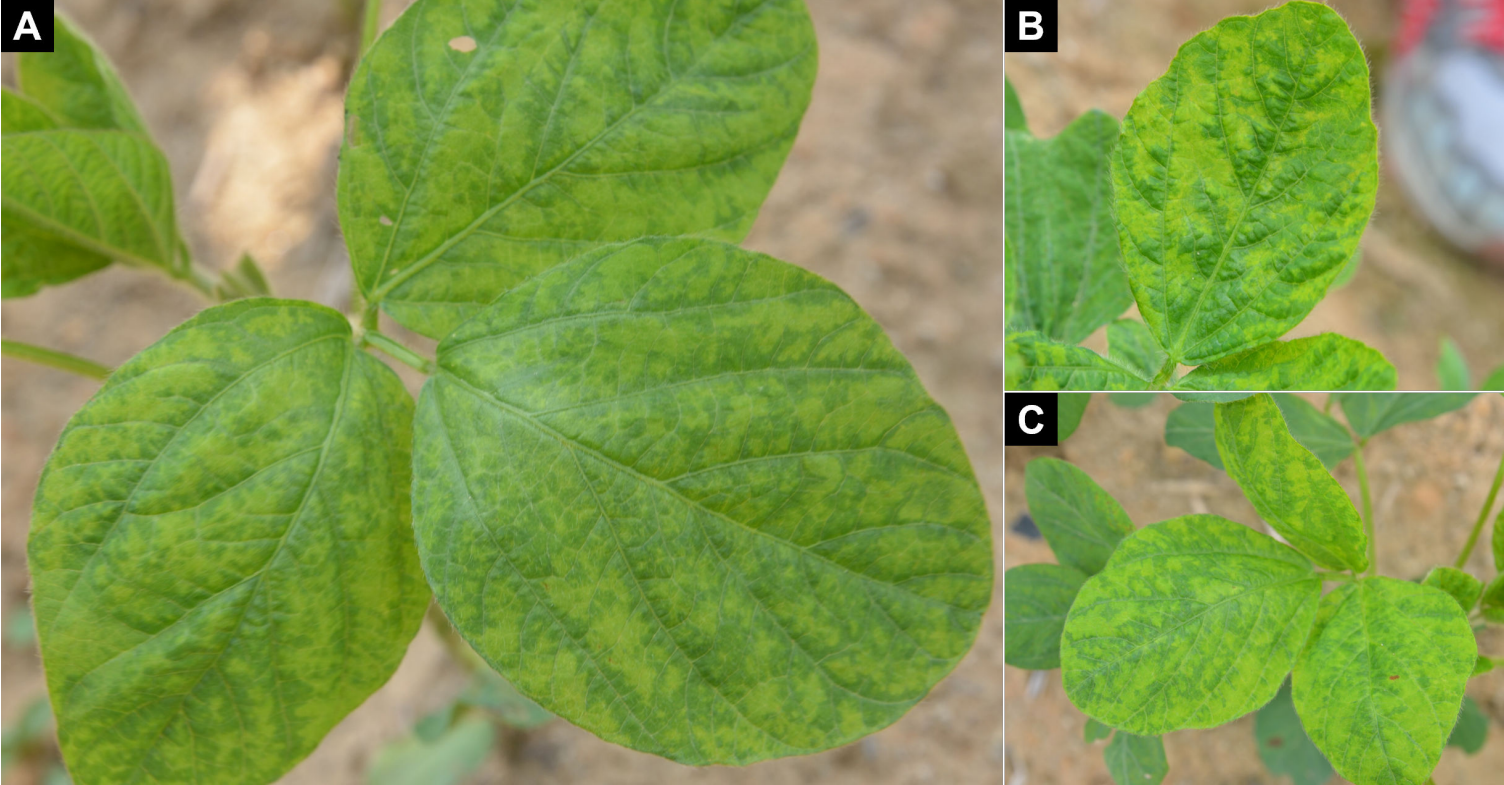
Soybeans infected with SYMMV exhibiting distinctive mosaic symptoms. Symptoms were observed in approximately 30% of plants in the same plot, and SYMMV infection was confirmed by RT-PCR assay. (A) Soybean plant in which a seed-transmitted isolate of SYMMV, ST-HB56, was detected. (B and C) Distinctive mosaic symptoms on the leaves.

In June 2020, distinct mosaic symptoms (Fig. 1) were observed in approximately 30% of early-stage soybeans cultivated at the experimental site of the National Institute of Crop Science in Daegu, Korea. Afterward, 14 leaves exhibiting this distinctive symptomatic feature were collected. An reverse transcription-polymerase chain reaction (RT-PCR) assay was conducted targeting 11 species of viruses, including alfalfa mosaic virus, bean common mosaic virus, clover yellow vein virus, cucumber mosaic virus, peanut stunt virus, peanut mottle virus, soybean dwarf virus, soybean mosaic virus, soybean yellow common mosaic virus, SYMMV, and tomato spotted wilt virus, following the methodology of a previous study (2). This inquiry definitively strongly suggested that the symptoms were related to an infection with SYMMV. Subsequently, SYMMV-infected plants were conventionally grown at the experimental site, and seeds were harvested from three infected plants. In a controlled greenhouse, 300 seeds were cultivated with 100 seeds per plant. Almost all of them successfully germinated, except for two seeds. Following a 4-week interval post-sowing, an RT-PCR assay was conducted using second trifoliolate leaves to determine the presence of SYMMV infection in the subsequent generation of soybean plants. As a result, the seed transmission of SYMMV was identified in a single soybean plant. To uncover the complete genome of the seed-transmitted SYMMV, total RNA was extracted from the second trifoliolate leaf of the corresponding soybean plant, in which vertical transmission was confirmed, by grinding the leaf using the easy-spin Total RNA Extraction Kit (iNtRON, Korea). Subsequently, cDNA was synthesized using random hexamers, and PCR was performed with six pairs of primers (Table 1) that were strategically designed to overlap segments of approximately 200–300 bp. Cloning was achieved using the All in One PCR Cloning Kit (Biofact, Korea), and subsequent sequencing was conducted with a Phred score ≥20 using the ABI 3730XL DNA Analyzer (Bioneer, Korea). The 5′- and 3′-RACE System for Rapid Amplification of cDNA Ends (Invitrogen, USA) with gene-specific primers (GSPs) (Table 1) enabled the determination of both termini, and the resulting raw sequences were assembled using the UGENE software (version 48.0). The complete genome sequence of the seed-transmitted SYMMV has a length of 4,008 bp, and five open reading frames (ORF) were predicted using the UGENE software (version 48.0) with default parameters. In an NCBI BLASTn search, it exhibited a 98.15% nucleotide identity with a 99% coverage compared to the GYGI-p isolate (MT603848.1). SYMMV, recently recognized as an emerging virus in Korean soybeans (2, 4, 5), remains relatively unexplored in terms of its specific ecological behaviors. The attributes of the seed-transmitted SYMMV isolates identified in this study are expected to contribute significantly to our understanding of SYMMV’s ecological dynamics.

**TABLE 1:** List of primer pairs designed to determine the complete genome sequence of seed-transmitted SYMMV^*a*^

Fragment	Oligo name	Sequence (5′ to 3′)	Expected size (bp)
1	F46	CGGAACAATGCCACCTGTCAG	863
	R908	CCCTCTCGATCAAACCTCTCCG	
2	F779	CTAATCACGCCTAGGAACGG	883
	R1661	GGAACTTCAGCCCAGACATG	
3	F1300	TGGACCCACTGTAATCAAGG	888
	R2187	AGTTTCTCCACAGCCACTTG	
4	F1891	CGTGTCAGGATGGAATAACG	856
	R2746	GTCGCTTTCGCTGGTTCTTGG	
5	F2437	GTGGCACATACGGTCACAGTC	859
	R3295	GCTGTCCATATGCTGCAACC	
6	F3095	GATCCAGACGCTAGTGATGC	687
	R3781	CATCGAGGTAGAGGGATTGTG	
5′-RACE^*b*^	R153	CCGAAAGCAGAGCAACAGTTG	–^*c*^
3′-RACE^*b*^	F3587	CTGCGGACTCCCCACTACTGATG	–^*c*^

^*a*^ Each primer pair was designed based on the SYMMV isolates registered in the NCBI GenBank.

^*b*^ These indicate the GSP used to determine both 5’ and 3’ termini.

^*c*^ The lengths of the 5' and 3' ends, which are untranslated regions, cannot be anticipated prior to the reaction.

## Data Availability

The complete genome sequence of seed-transmitted SYMMV isolate has been deposited at GenBank under accession number OP490296 .1. The raw sequence of Sanger reads has been deposited in the Sequence Read Archive under Bio Project accession number PRJNA1012842. Additionally, the sequence of the coat protein gene of the original virus preceding seed transmission is available under accession number MW079208 .1.
